# An Enhanced Strategy for Daily Disinfection in Acute Care Hospital Rooms: A Randomized Clinical Trial

**DOI:** 10.1001/jamanetworkopen.2022.42131

**Published:** 2022-11-15

**Authors:** Bobby G. Warren, Aaron Barrett, Amanda Graves, Carly King, Nicholas A. Turner, Deverick J. Anderson

**Affiliations:** 1 Division of Infectious Diseases, Duke Center for Antimicrobial Stewardship and Infection Prevention, Durham, North Carolina; 2 Disinfection, Resistance and Transmission Epidemiology (DiRTE) Lab, Duke University School of Medicine, Durham, North Carolina; 3 Division of Infectious Diseases, Duke University School of Medicine, Durham, North Carolina

## Abstract

**Importance:**

Environmental contamination is a source of transmission between patients, health care practitioners, and other stakeholders in the acute care setting.

**Objective:**

To compare the efficacy of an enhanced daily disinfection strategy vs standard disinfection in acute care hospital rooms.

**Design, Setting, and Participants:**

This randomized clinical trial (RCT) was conducted in acute care hospital rooms at Duke University Hospital in Durham, North Carolina, from November 2021 to March 2022. Rooms were occupied by patients with contact precautions. Room surfaces (bed rails, overbed table, and in-room sink) were divided into 2 sides (right vs left), allowing each room to serve as its own control. Each side was randomized 1:1 to the intervention group or control group.

**Interventions:**

The intervention was a quaternary ammonium, salt-based, 24-hour continuously active germicidal wipe. It was applied in addition to routine disinfection for the intervention group. The control group received no intervention beyond routine disinfection.

**Main Outcomes and Measures:**

The primary outcome was the total contamination, measured in colony-forming units (CFUs) on the bed rails, overbed table, and sink on study day 1. The secondary outcomes were the proportion of sample areas with positive test results for clinically important pathogens, including methicillin-resistant *Staphylococcus aureus*, vancomycin-resistant *Enterococcus,* and carbapenem-resistant *Enterobacteriaceae*; the similarity in baseline contamination between sample area sides on study day 0 before application of the intervention, and the proportion of sample areas with removed UV luminescent gel on study day 1*.*

**Results:**

A total of 50 study rooms occupied by 50 unique patients (median [IQR] age, 61 [45-69] years; 26 men [52%]) with contact precautions were enrolled. Of these patients, 41 (82%) were actively receiving antibiotics, 39 (78%) were bedridden, and 28 (56%) had active infections with study-defined clinically important pathogens. On study day 1, the median (IQR) total CFUs for the intervention group was lower than that for the control group (3561 [1292-7602] CFUs vs 5219 [1540-12 364] CFUs; *P* = .002). On study day 1, the intervention side was less frequently contaminated with patient-associated clinically important pathogens compared with the control side of the room (4 [14%] vs 11 [39%]; *P* = .04).

**Conclusions and Relevance:**

Results of this RCT demonstrated that a quaternary ammonium, salt-based, 24-hour continuously active germicidal wipe decreased the environmental bioburden in acute care hospital rooms compared with routine disinfection. The findings warrant large-scale RCTs to determine whether enhanced daily disinfection strategies can decrease patient acquisition and adverse patient outcomes.

**Trial Registration:**

ClinicalTrials.gov Identifier: NCT05560321

## Key Points

**Question:**

How does a quaternary ammonium, salt-based, 24-hour continuously active germicidal wipe compare with standard disinfection in acute care hospital rooms?

**Findings:**

In this randomized clinical trial of inpatient rooms occupied by 50 unique patients, the median total bioburden for the intervention group was statistically significantly lower than that for the control group on study day 1. Enhanced daily disinfection decreased the environmental bioburden in acute care hospital rooms compared with routine disinfection.

**Meaning:**

Findings of this study support performing large-scale randomized clinical trials to ascertain whether enhanced daily disinfection strategies can decrease patient acquisition of infection and adverse patient outcomes.

## Teaser

This randomized clinical trial compares the efficacy of a quaternary ammonium, salt-based, 24-hour continuously active germicidal wipe vs routine disinfection in inpatient rooms.

## Introduction

Approximately 721 000 health care–associated infections (HAIs) occur each year in the US, and approximately 75 000 patients die in the hospital as a result.^1,2^ Many HAIs are caused by multidrug-resistant organisms, which occur in 2 million people in the US each year and lead to adverse patient outcomes and mortality.^2,3,4,5^

Pathogens that cause HAIs are frequently found on health care surfaces, textiles, and sinks.^6,7,8,9^ There is increasing evidence that pathogens found in the health care environment also play a role in transmission between patients, health care practitioners, and other stakeholders.^10,11,12,13^ As a result, hospital surface disinfection is key to preventing HAIs and pathogen transmission. However, several important challenges prevent effective disinfection through routine chemical disinfection application, such as compliance, thoroughness, and duration of disinfectant activity; thus, enhanced strategies for disinfection are needed.^14,15,16^

In general, US Environmental Protection Agency–registered liquid chemical disinfectants used in health care settings are highly effective. Disinfection occurs immediately after disinfectant application, but surfaces quickly become recontaminated. Thus, one potential solution to decrease the risk of transmission through the environment is continuous disinfection using technologies that have constant or long-lasting disinfectant action as opposed to only when applied.

We performed a randomized clinical trial (RCT) to determine the efficacy of an enhanced daily disinfection strategy compared with standard disinfection in acute care hospital rooms. The disinfectant we examined has demonstrated efficacy immediately after application and for 24 subsequent hours.^17^ Our a priori hypothesis was that the use of this enhanced disinfection technology compared with routine disinfection would decrease the environmental bioburden in hospital rooms.

## Methods

The Duke University Health System Institutional Review Board deemed this RCT exempt because no intervention was performed on patients. The institutional review board also granted a waiver of informed patient consent to enable access to patient medical records to identify study participants. The protocol is provided in Supplement 1. We followed the Consolidated Standards of Reporting Trials (CONSORT) reporting guideline.

The primary objective was to compare the efficacy of Sani-24 (PDI Healthcare), a quaternary ammonium, salt-based, 24-hour continuously active germicidal wipe with the standard disinfection strategy. The secondary objectives were to assess a novel sampling strategy to resolve case-mix issues with environmental sampling and to describe routine disinfection compliance.

### Study Design and Setting

We performed this RCT in acute care hospital rooms at Duke University Hospital, a 1048-bed tertiary care hospital in Durham, North Carolina, from November 2021 to March 2022. All inpatient rooms occupied by adult patients (18 years or older) were eligible for enrollment. Study rooms were identified by routine review of bed flow data. Rooms occupied by patients with contact precautions were targeted for inclusion. Rooms were excluded if the patient was discharged before samples from study day 0 and day 1 were obtained.

Three areas in each study room were split in half for the purposes of this study: bed rails (left vs right), overbed table (left vs right), and in-room sink (left vs right). Sample area sides in each room were randomized (1:1) to the intervention group or control group by a flip of a coin (Figure). Thus, overall, we evaluated a total of 3 sample areas that were split into 2 sides, with 1 side of each area included in each study group. All of the clinical, laboratory, and statistical staff were blinded to the randomization (ie, triple-blind study); however, the study team members (including A.B.) who applied the disinfectant and collected environmental cultures were not blinded to the study group randomization.

**Figure.**
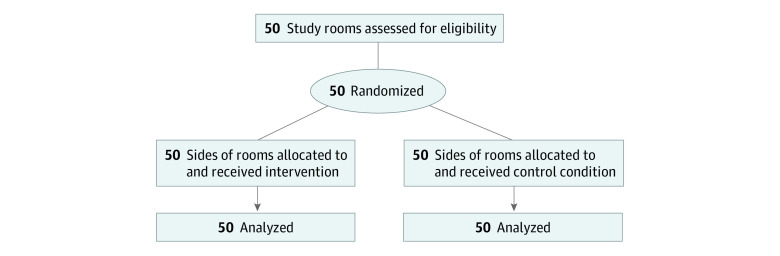
CONSORT Flow Diagram

### Study Protocol

In areas in the intervention group, a study team member applied the enhanced disinfectant and allowed the area to air-dry according to the manufacturer’s instructions for use (study day 0) in addition to routine disinfection. The areas in the control group received no intervention beyond routine disinfection. The environmental services (EVS) team was blinded to the study group randomization or study activities and was allowed to perform routine disinfection per standard hospital protocols. All study rooms were occupied by patients with contact precautions; thus, the EVS team performed terminal disinfection using bleach and UV-C as well as daily disinfection using a bleach-based solution.

On study day 0, the study team also placed a UV light–induced luminescent gel (Glo Germ; Glo Germ Company) on each sample area to evaluate routine disinfection compliance. Environmental cultures were taken on study day 0 immediately before the application of the intervention or application of the UV luminescent gel and on study day 1 approximately 24 hours after application of the intervention. Cultures were obtained for both study groups from all 3 locations within each study room on day 0 and day 1.

### Microbiological Methods

All environmental cultures were obtained directly from the clinical environment using the sponge and stomacher technique in accordance with the Centers for Disease Control and Prevention protocol.^18^ Sponges were premoistened with 10 mL of Dey-Engley neutralizing buffer before culture collection. After collection, sponges were placed in stomacher bags with 45 mL of phosphate-buffered saline with 1% Tween 20 and then homogenized for 60 seconds at 260 rpm. Homogenates were then centrifuged at 3200 rpm for 15 minutes, and all but approximately 5 mL of the resulting supernatant was discarded. Next, each culture was rehomogenized via vortex. A total 100 μL of the final homogenate was plated onto trypticase soy agar with 5% sheep’s blood for overall bioburden; 200 μL of the homogenate was plated onto selective media: bile esculin agar for *Enterococcus* spp, mannitol salt agar for *Staphylococcus aureus*, and MacConkey agar for gram-negative species of interest. The presence of *Enterococcus spp* and *S aureus* were confirmed using standard laboratory procedures, and gram-negative bacteria were speciated using matrix-assisted laser desorption ionization time-of-flight mass spectrometry.

For this study, we defined clinically important pathogens as methicillin-resistant *S aureus* (MRSA), vancomycin-resistant *Enterococcus* (VRE), and carbapenem-resistant *Enterobacteriaceae* (CRE). The presence of antibiotic resistance genes was determined via polymerase chain reaction test results for MRSA (mecA [GenBank X52593]), VRE (VanA [GenBank M97297]), and CRE (KPC [GenBank AF297554], NDM-1 [GenBank JF703135], VIM [GenBank AF191564], OXA48 [GenBank AY236073], and IMP [GenBank AJ584652]).

### Outcomes

The primary outcome was the total contamination, measured in colony-forming units (CFUs), on environmental surfaces on study day 1. Results from the intervention group were compared against results from the control group. Total room CFUs were determined by adding the CFUs detected by individual cultures in 1 study group to yield a single value (eg, results from cultures from all 3 right surfaces vs all 3 left surfaces). The secondary outcomes were the proportion of sample areas with a positive result for individual clinically important pathogens, the similarity in baseline contamination between sample area sides on study day 0 before application of the intervention, and the proportion of sample areas with removed UV luminescent gel on study day 1.

### Statistical Analysis

The Wilcoxon signed rank test was used to compare all CFU measurements, and the *z* score proportionality test was used to compare proportions of sample areas with clinically important pathogens and with removed UV luminescent gel. *P* < .05 was considered to be significant. All statistical tests were 2-tailed and performed using SAS, version 9.4M7 (SAS Institute Inc).

## Results

From November 2021 to March 2022, a total of 50 study rooms were analyzed (Figure). These rooms were occupied by 50 unique patients (median [IQR] age, 61 [45-69] years; 24 women [48%] and 26 men [52%]) with contact precautions. Of these patients, 41 (82%) were actively receiving antibiotics, 39 (78%) were bedridden, and 28 (56%) had active infections with study-defined clinically important pathogens (Table 1).

**Table 1.** Patient Characteristics

Characteristic	Patients, No. (%) (N = 50)
Sex	
Female	24 (48)
Male	26 (52)
Age, median (IQR), y	61 (45-69)
Hospitalized in past 90 d	23 (46)
Contact isolation	50 (100)
Length of stay at enrollment, median (IQR), d	20 (9-57)
Length of stay in study room at enrollment, median (IQR), d	6 (3-15)
Active infection	50 (100)
Blood	16 (32)
Urine	12 (24)
Tissue	8 (16)
Respiratory	9 (18)
Other	7 (14)
Active infection with study-defined clinically important pathogens	28 (56)
Blood	10 (20)
Urine	5 (10)
Tissue	7 (14)
Respiratory	4 (8)
Other	2 (4)
Active antibiotic therapy	41 (82)
Patient function	
Bedridden	39 (78)
Rectal tube	2 (4)
Catheter	34 (68)
None	6 (12)
Aerosol-generating procedures	
Intubation	19 (38)
Bronchoscopy	1 (2)
None	31 (62)

A total of 600 environmental cultures were obtained, of which 300 were from each study group and 200 were from each sample area. The total sample area in study rooms was 4150 cm^2^, representing 2000 cm^2^ from the bed rail, 1750 cm^2^ from the overbed table, and 400 cm^2^ from the in-room sink. Thus, each study group contributed 2075 cm^2^, representing 1000 cm^2^ from the bed rail, 875 cm^2^ from the overbed table, and 200 cm^2^ from the in-room sink.

The median (IQR) total CFUs per study room was 4692 (2107-10 290). The median (IQR) total was 5319 (2627-10 289) CFUs on study day 0 and 3912 (1488-10 989) CFUs on study day 1. Contamination was found on all tested surfaces. A total of 142 of 600 clinically important pathogens (24%) were found in environmental cultures in all study rooms, of which 74 (12%) were found on study day 0 and 68 (11%) on study day 1 (Table 2).

**Table 2.** CFUs, Clinically Important Pathogens, and UV Luminescent Gel Removal

Variable	Both study days, total	Study day 0			Study day 1		
		Intervention group	Control group	*P* value	Intervention group	Control group	*P* value
Room CFUs, median (IQR)	4692 (2107-10 290)	4851 (2574-9617)	6059 (2629-13 175)	.62	3561 (1292-7602)	5219 (1540-12 364)	.002
Bed rails	919 (186-3076)	1067 (262-2919)	909 (216-2674)	.79	876 (113-3141)	883 (198-4090)	.19
Overbed table	1221 (396-3281)	1257 (407-3393)	1474 (450-4163)	.54	710 (390-2588)	1278 (318-4002)	.34
In-room sink	891 (280-2400)	1288 (438-2927)	1191 (485-2598)	.62	320 (85-996)	1102 (344-2946)	<.001
Total clinically important pathogens, No./total No. (%)	142/600 (24)	35/150 (23)	39/150 (26)	.59	29/150 (19)	39/150 (26)	.17
Bed rails	43/200 (22)	10/50 (20)	9/50 (18)	.80	13/50 (26)	11/50 (22)	.64
Overbed table	49/200 (25)	14/50 (28)	13/50 (26)	.82	7/50 (14)	15/50 (30)	.05
In-room sink	50/200 (25)	11/50 (22)	17/50 (34)	.18	9/50 (18)	13/50 (26)	.33
Total UV luminescent gel removed from sample areas	NA	NA	NA	NA	16/150 (11)	17/150 (11)	.86
Bed rails	NA	NA	NA	NA	3/50 (6)	4/50 (8)	.70
Overbed table	NA	NA	NA	NA	6/50 (12)	6/50 (12)	>.99
In-room sink	NA	NA	NA	NA	7/50 (14)	7/50 (14)	>.99

Abbreviations: CFUs, colony-forming units; NA, not applicable.

At study day 0 (baseline), the median (IQR) total CFU for the intervention group was 4851 (2574-9617), whereas the median (IQR) total CFU for the control group was 6059 (2629-13 175) (*P* = .62). On study day 1, the median (IQR) total CFU for the intervention group was lower than that for the control group (3561 [1292-7602] CFUs vs 5219 [1540-12 364] CFUs; *P* = .002) (Table 2). In addition, when comparing room CFUs on study day 0 vs study day 1, room CFUs for the control group were similar between study days (6059 [2629-13 175] CFUs vs 5219 [1540-12 364] CFUs; *P* = .87), and the room CFUs for the intervention group were significantly lower on study day 1 (4851 [2574-9617] CFUs vs 3561 [1292-7602] CFUs; *P* = .04). All sample area sides for both study groups were contaminated with flora.

At study day 0, the median CFUs were similar for intervention and control groups (Table 2). However, differences per location were observed on study day 1, particularly at the sink. On study day 1, the median (IQR) CFUs were lower at each location in the intervention group compared with the control group (bed rails: 876 [113-3141] CFUs vs 883 [198-4090] CFUs; *P* = .19; overbed table: 710 [390-2588] CFUs vs 1278 [318-4002] CFUs; *P* = .34; and sink: 320 [85-996] CFUs vs 1102 [344-2946] CFUs; *P* < .001).

Overall, isolation of clinically important pathogens was not different among the intervention and control groups at study day 0 or day 1 (Table 2). However, important differences were observed when the evaluation was limited to patients with known infection or colonization with clinically important pathogens (Table 3). Of 50 patients, 28 (56%) had active infections from study-defined clinically important pathogens, and 19 (68%) occupied rooms were contaminated with the same clinically important pathogens that the patients had. Among these 28 patients at study day 0, the intervention side of the room was contaminated with a similar number of patient-associated clinically important pathogens compared with the control side (15 [54%] vs 10 [36%]; *P* = .18). On study day 1, the intervention side of the room was less frequently contaminated with patient-associated clinically important pathogens compared with the control side (4 [14%] vs 11 [39%]; *P* = .04). These patterns were also seen at the sample area level, with similar values between the intervention and control groups on study day 0 (21 [25%] vs 19 [23%]; *P* = .72) and less contamination in the intervention group on study day 1 (9 [11%] vs 20 [24%]; *P* = .02) (Table 3).

**Table 3.** Environmental Contamination With Patient-Associated Clinically Important Pathogens^a^

Variable	Clinically important pathogens detected, No. (%)						
	Both study days, total	Study day 0			Study day 1		
		Intervention group	Control group	*P* value	Intervention group	Control group	*P* value
Room clinically important pathogens (n = 28)	19 (68)	15 (54)	10 (36)	.18	4 (14)	11 (39)	.04
Total clinically important pathogens^b^	69 (21)	21 (25)	19 (23)	.72	9 (11)	20 (24)	.02
Bed rails^b^	18 (15)	5 (18)	6 (21)	.73	3 (11)	4 (14)	.69
Overbed table^b^	27 (24)	10 (36)	5 (18)	.13	3 (11)	9 (32)	.05
In-room sink^b^	24 (21)	6 (21)	8 (29)	.54	3 (11)	7 (25)	.16

^a^ Patient-associated clinically important pathogens are found in study rooms wherein the patient had an active infection with the same species.

^b^ Total number of clinically important pathogens changed for each study day and study group.

Compliance with daily disinfection by the EVS team was low during the study period. Overall, 13 of 50 study rooms (26%) had 1 or more UV luminescent gel removed, and only 2 study rooms (4%) had all 3 UV luminescent gel removed from sample area sides. At the sample area level, 33 of 300 sides (11%) had UV luminescent gel removed from their surface between study days 0 and 1 (7 from bed rails, 12 from overbed tables, and 14 from sinks). Overall, the total UV luminescent gel removed was comparable between the control and intervention groups (bed rails: 4 [8%] vs 3 [6%]; *P* = .70; overbed table: 6 [12%] vs 6 [12%]; *P* > .99; and sink: 7 [14%] vs 7 [14%]; *P* > .99) (Table 2).

## Discussion

Data confirming the role that the health care environment plays in the transmission of clinically important pathogens continue to increase. Standard disinfection is effective if and when applied correctly, but its maximal effect is quickly reduced due to important and ongoing challenges, such as improper use of disinfectant and low compliance with disinfection strategy.^19,20,21,22^ Thus, enhanced strategies for disinfection are needed. To our knowledge, this RCT was the first to investigate the efficacy of an enhanced disinfection strategy for daily disinfection in acute care hospital rooms. 

In this trial, enhanced disinfection led to a significant decrease in total room CFUs on study day 1 compared with study day 0 as well as a decrease in the number of patient-associated clinically important pathogens found in the environment on study day 1. Contamination was lower in all 3 sample areas in the intervention group compared with the control group; however, the statistical significance appeared to be driven primarily by the sink, and thus the results should be interpreted with that finding in mind. These findings indicate that this enhanced disinfection technology was efficacious at reducing bioburden in acute care hospital rooms and, as a result, could be used as a strategy to decrease risk of HAI in hospitalized patients.

Few studies have evaluated enhanced disinfection strategies using randomization. Results of the present trial are different from results of previous RCTs that demonstrated a lack of efficacy or extreme efficacy of enhanced continuously active, quaternary ammonium, salt-based disinfectants.^9,23,24,25^ For example, Warren et al^9^ found that use of a persistent quaternary ammonium organosilane compound had no efficacy for microbial load in outpatient clinics, and Boyce et al^25^ found no efficacy for high-touch surfaces in study rooms. Schmidt et al^23^ demonstrated a decrease in efficacy and continuous activity of 2 similar products at 1 hour and 6 hours after application, and Tahmimi et al^24^ demonstrated continuous efficacy (>2 log reduction) of up to 8 weeks after application. These differences in study results are likely related to the length of expected disinfectant activity (Warren et al^9^), varying sampling techniques: contact plates vs sponge and stomacher (Boyce et al^25^), experimental vs clinical environment (Schmidt et al^23^), and location of sample areas (Tahmimi et al^24^). Contact plates can sample only 25 cm^2^ and can miss or hit areas of higher contamination due to the heterogeneity of contamination. In contrast, the sponge and stomacher method can sample a much larger surface area, resulting in a mean bioburden. In addition, using the sponge and stomacher method to sample small areas, such as 100 cm^2^, can be subject to the heterogeneity of environmental contamination; repeated sampling of these small areas reduces contamination, giving a manufactured bioburden reduction.^24^

In contrast, results of the present RCT are similar to previous nonrandomized studies, such as the study by Garvey et al,^26^ which demonstrated the effectiveness of a quaternary ammonium, salt-based wipe in reducing MRSA acquisition in a large teaching hospital, and the study by Kopp et al,^27^ which demonstrated the efficacy of another quaternary ammonium, salt-based wipe in reducing the bioburden of personal devices of critical care nurses.

The present study used a novel environmental sampling strategy to resolve case-mix issues. We assessed the efficacy of the enhanced disinfectant by comparing study groups within study rooms occupied by 1 patient and splitting the large sample areas in half as opposed to enrolling different study rooms in each group. This approach led to similar baseline overall and clinically important pathogen contamination levels on study day 0 and allowed us to better conclude that the differences observed on day 1 were related to products under evaluation rather than to differences among rooms or patients.

We described routine disinfection compliance in study rooms by measuring UV luminescent gel removal. Briefly, UV luminescent gel removal was minimal overall (26% room level and 11% sample level) and was similar between control and intervention groups (11% for both). Even if we assumed that removal of UV luminescent gel occurred because of EVS disinfection (as opposed to physical removal for some other reason), these data suggest that routine disinfection compliance was low in all study rooms and was equally represented in each study group.

### Limitations

This study has limitations. First, the disinfection of clinically relevant spore-forming bacteria was not assessed given that quaternary ammonium compounds are not efficacious for these pathogens. Second, only rooms occupied by patients with contact precautions were enrolled. This eligibility requirement may have set a higher bioburden baseline on day 0 compared with noncontact rooms. Moreover, 78% of patients were bedridden, which may have affected the environmental contamination of sample areas, such as inflating bed rail and overbed table contamination as well as deflating sink contamination. Third, enhanced disinfectant was applied by the study team, and most patients were in intensive care units. As a result, generalizability to other settings may be limited. Fourth, patient acquisition and clinical outcomes were not measured.

## Conclusions

After ensuring adequate patient case-mix through a novel environmental sampling strategy, this trial demonstrated that a quaternary ammonium, salt-based, 24-hour continuously active germicidal wipe decreased the environmental bioburden in acute care hospital rooms compared with routine disinfection. Daily disinfection otherwise occurred infrequently during the study. The findings support performing large-scale RCTs to determine whether enhanced daily disinfection strategies can decrease patient acquisition and adverse patient outcomes. Future studies may use this sampling strategy, particularly in clinical settings using RCT methods.

## Notes

Supplement 1.
Trial Protocol

Supplement 2.
Data Sharing Statement
