# Supplementary material for: An Enhanced Strategy for Daily Disinfection in Acute Care Hospital Rooms: A Randomized Clinical Trial
Source: JAMA Netw Open. 2022 Nov 15;5(11):e2242131. doi: 10.1001/jamanetworkopen.2022.42131 (PMC9667331; doi:10.1001/jamanetworkopen.2022.42131)
Supplement: Supplement 1. — Trial Protocol [file jamanetwopen-e2242131-s001.pdf]

DUHS IRB Application (Version 1.1)

General Information

***Please enter the full title of your protocol:**

Measuring the Impact of an Enhanced Strategy for Daily Disinfection in Acute Care Hospital Rooms

***Please enter the Short Title you would like to use to reference the study:**

Measuring the Impact of an Enhanced Strategy for Daily Disinfection in Acute Care Hospital Rooms
* This field allows you to enter an abbreviated version of the Study Title to quickly identify this study.

Add Study Organization(s):

List Study Organizations associated with this protocol:

Primary Dept?	Department Name
◆	**DUHS** - Medicine-Infectious Diseases

Assign key study personnel (KSP) access to the protocol

*** Please add a Principal Investigator for the study:**

(Note: Before this study application can be submitted, the PI MUST have completed CITI training)

Sexton, Daniel

3.1 **If applicable, please select the Key Study personnel: (Note: Before this study application can be submitted, all Key Personnel MUST have completed CITI training)**
*** Denotes roles that are not recognized in OnCore. Please select an appropriate role that is recognized in all clinical research applications (iRIS, OnCore, eREG, etc.)**

A) Additional Investigators, Primary Study Coordinator (CRC), and the Primary Regulatory Coordinator (PRC):

Nelson, Alicia
Primary Study Coordinator (CRC/CRNC/RPL)
Nelson, Alicia
Primary Regulatory Coordinator

B) All Other Key Personnel

Addison, Rachel
Study Coordinator (CRC/CRNC/RPL)

Anderson, Deverick
Sub-Investigator
Foy, Katherine
Study Coordinator (CRC/CRNC/RPL)
Warren, Bobby
Sub-Investigator

***Please add a Study Contact:**

Nelson, Alicia
Sexton, Daniel
Warren, Bobby

The Study Contact(s) will receive all important system notifications along with the Principal Investigator. (e.g., The study contact(s) are typically the Principal Investigator, Study Coordinator, and Regulatory Coordinator.)

Oncore

Please select the Library for your Protocol:

This field is used in OnCore. Determines the Reference Lists, Forms, Protocol Annotations, Notifications, and Signoffs available for the protocol. Protocols that require reporting to the NCI (National Cancer Institute), must select the Oncology library.

- ○ Oncology
- ◉ Non-Oncology

Protocol Application Type

Select the type of protocol you are creating:

Please see additional criteria and information in the policy titled "Reliance on the IRB of Another Institution, Organization, or an Independent IRB" on the **IRB web site**.

- ○ Regular Study Application - Most common. The IRB will determine if the study is eligible for expedited review or requires full board review upon submission.
- ◉ Application for Exemption from IRB Review - Includes Exempt, Not Human Subject Research, & Not Research.
- ○ External IRB Application - Any study using an external IRB as the IRB-of-Record.
- ○ Trainee Research While Away from Duke - Research conducted by medical students overseen by the Office of Curriculum & other student/trainee research away from Duke.
- ○ Individual Patient Expanded Access, Including Emergency Use - Use of an investigational product under expanded access, including emergency use of an investigational drug or biologic or emergency use of an unapproved device.

Oversight Organization Selection

CRU (Clinical Research Unit) or Oversight Organization Selection:

Please select the CRU.

Medicine

The Clinical Research Unit that takes responsibility for this study.

- Please select **Medicine** as the CRU **only** if the PI is in one of these Divisions or Institutes: Endocrinology, Gastroenterology, General Internal Medicine, Geriatrics, Hematology, Infectious Diseases, Nephrology, Pulmonary, Rheumatology & Immunology, Center for Applied Genomics and Precision Medicine, Center for the Study of Aging and Human Development, Duke Molecular Physiology Institute.
- More information on CRUs can be found on the Duke Office of Clinical Research (DOCR) website, **http://docr.som.duke.edu**
- Questions concerning CRU selection should be directed to docr.help@dm.duke.edu.
- For questions about the Campus Oversight Organization, please visit **Campus Oversight Organization**.

List all Key Personnel on the study who are outside Duke:

- **Note:** You will also need to attach the documentation of Human Subjects Certification for each individual, if they have completed the certification somewhere other than Duke.
- **If outside key personnel will have access to Duke PHI, a data transfer agreement AND external site IRB approval (or IRB authorization agreement) will be needed.** See HRPP policy **Use of Research Data by Former Duke Students or Former Duke Faculty and Employees**
- In the panel below, "PHI" is Protected Health Information.

Entry 1

**Name**	
**Study Role**	
**Email Address**	
**Institution / Organization**	
**Will he/she have access to Duke P.H.I.?**	○ Yes ○ No
**Is he/she an unpaid volunteer at Duke on the study?**	○ Yes ○ No

Sponsor and Funding Source

Add all funding sources for this study:

View Details	Sponsor Name	Sponsor Type	Contract Type:	Project Number	Award Number
⊟	Duke University	Institutional			

Sponsor Name:	Duke University
Sponsor Type:	Institutional
Sponsor Role:	Funding
**Grant/Contract Number:**	
**Project Period:**	From: to:
**Is Institution the Primary Grant Holder:**	No
**if No, then who is the Primary Grantee?**	
Contract Type:	

Project Number:	
Award Number:	
Grant Title:	
PI Name:<br>(If PI is not the same as identified on the study.)	
Explain Any Significant Discrepancy:	

Is this a federally funded study?

○ Yes ◉ No

Does this study have any of the following?

- Industry sponsored protocol
- Industry funded Duke protocol
- Industry funded sub-contract from another institution
- Industry provided drug/device/biologic
- SBIR/STTR funded protocol

○ Yes ◉ No

As part of this study, will any samples or PHI be transferred to/from Duke to/from anyone other than the Sponsor, a Sponsor subcontractor, or a Funding Source?

○ Yes ◉ No

Is the Department of Defense (DOD) a funding source?

○ Yes ◉ No

Have you successfully synced your protocol to OnCore by clicking the 'Sync Data Over API' button at the top of this page?

Please verify that the protocol has been created in OnCore before submitting this application for PI Signoff.

◉ Yes, I synced my protocol to OnCore and verified it was successfully sent by logging into OnCore.
○ I may have forgotten! I'll click it again right now, just to be sure, and verify it was successfully sent by logging into OnCore.

Mobile Devices and Software

Does this study involve the use of a software or a mobile application?

○ Yes ◉ No

List all software, including third party (non-Duke) and mobile apps, that will be utilized for ascertainment, recruitment, or conduct of the research/project: (eg, MaestroCare, DEDUCE):

Exempt Application

Project Summary:

Note: Data generated from an exempt project cannot be used in support of an FDA application for a drug/device/biologic. If you plan to utilize this data in support of an FDA application, please do not continue with this exemption request and, instead, submit using the Regular Study Application pathway.

*Will you use data generated from this project to support an FDA application for a drug/device /biologic?.

○ Yes ◉ No

State the objectives of the research/project:

To determine the effectiveness of an enhanced strategy for daily disinfection in acute care hospital rooms comparing the addition of sani24 to routine daily cleaning versus the control of routine daily cleaning. We will evaluate the effectiveness of a) adding sani24 to the standard daily cleaning and b) routine daily disinfection in acute care hospital rooms to measure the reduction in bioburden. In other words, we aim to answer the following research question: *does the addition of an additional disinfection technology (Sani24) decrease the environmental bioburden in inpatient hospital compared to routine disinfection*?

Briefly describe research/project activities occurring at Duke, including the source of all data /samples:

- Do not copy and paste an external entity's summary. Please limit to 3 paragraphs.

Design – randomized controlled trial.

Setting – we will perform this study in the acute care floors of the Duke University Health System:

Study arms – This trail will include two study arms:

1. Control – routine, standard disinfection strategies will be used throughout the study.
2. Active Study Arm 1 – Sani24 will be sprayed on three standard high touch areas by study staff at the same point each morning in addition to routine daily disinfection.

Intervention – Two acute care patient rooms in similar units will be chosen that meet the following criteria:

1. Rooms from which a patient on contact precautions was discharged.
2. Rooms housing recently admitted patients with a projected length of stay >=3 days.

Chosen similar rooms will be paired into study couplets. One room will be randomized to intervention or control arm and its pair will be assigned the opposite arm. Hospital personnel and patients will be blinded to the study arm.

Those randomized to intervention rooms will receive applications of Sani24 immediately following enrollment and the successive 2-days in addition to routine daily disinfection. Those randomized to control will receive only routine daily disinfection.

Procedure – We will obtain environmental cultures from each study couplet's room immediately following their enrollment on hospital day 0, and on hospital day 3. Environmental cultures will be obtained using the swab and stomacher technique, per CDC protocols. Cultures will be obtained from three locations: Bedrails, the overbed table and the sink.

Data Collection – Total colony forming units (CFU) will be calculated from each culture and aggregated for total room CFU. Culture specimens will be evaluated for specific pathogens: *S. aureus* (MRSA or MSSA), *Enterococci* (VRE or VSE), *Acinetobacter* spp., *Pseudomonas* spp., and *Enterobacteriaceae* of interest such as *E. coli* and *Klebsiella* spp.

We will not collect PHI during this procedure but will need to access medical records (please see *Re quest for Waiver or Alteration of Informed Consent*) for limited PHI on the patient in each room, specifically microbiological cultures. We will also determine if any of those patients had an active or history of infection or colonization with one of our organisms of interest.

Attach all documents such as questionnaires, surveys, scripts and/or agreements in the Initial Review Submission Packet.

Are you planning to consent subjects?

○ Yes ◉ No

Will PHI be accessed for ascertainment or recruitment?

◉ Yes ○ No

Will PHI be utilized without consent for the conduct of the research/project?

◉ Yes ○ No

Target Enrollment:

Number of consented subjects:

- Enter a single number. If you anticipate consenting a range of subjects, enter the upper limit of the range. The number should represent the maximum number of subjects for the life of the study.

0

Number of individuals whose data/samples will be used:

150

Describe how research data will be stored and secured to ensure confidentiality:

Data will be stored on encrypted Duke Medicine servers (participant log & study IDs) and/or in our REDCap database (all other data collected for the study). No PHI will be collected as part of this study. Clinic rosters will be reviewed each day to determine the number with infection or colonization with a multidrug-resistant organism.

Describe how research data will be collected and/or transmitted during the research/project (ie, survey results entered by subjects, data transmitted to study team, data emailed to external sites).

Include information about the security of networks and any third parties that may be involved.

Total colony forming units (CFU) will be calculated from each culture and aggregated for total room CFU. Culture specimens will be evaluated for specific pathogens: S. aureus (MRSA or MSSA), Enterococci (VRE or VSE), Acinetobacter spp., Pseudomonas spp., and Enterobacteriaceae of interest such as E. coli and Klebsiella spp.
We will not collect PHI during this procedure but will need to access medical records (please see Request for Waiver or Alteration of Informed Consent). We will also determine if any of those patients had an active or history of infection or colonization with one of our organisms of interest. However, only aggregate data will be recorded.

Waiver of Consent and HIPAA Authorization

Attach your Request for Waiver or Alteration of Consent and HIPAA Authorization in the Initial Submission Packet.

- **Note:** If you are applying for an exemption under QI/QA, a waiver might not be required. If you have questions, please consult with the IRB.

Request for Waiver or Alteration of Consent and/or HIPAA Authorization

Will the population include deceased individuals?

○ Yes ◉ No

This waiver request applies to the following research activity or activities:

☐ Scheduling of research activities in MaestroCare and/or the recording of PHI via telephone for screening purposes prior to obtaining written consent for the research. Scheduling of research activities in MaestroCare and/or the recording of PHI via telephone for screening purposes prior to obtaining written consent for the research. (If you check this box, please complete all sections below.)

☐ Ascertainment (identification, selection) and/or recruitment of potential subjects while recording identifiable private information, such as protected health information (PHI), prior to obtaining the subject’s consent. (If you check this box, please complete sections B and C below.)

☑ Conduct of the research project without obtaining verbal or written consent and authorization. (If you check this box, please complete sections B and C below.)

Provide the following information:

List the elements of informed consent and/or HIPAA authorization for which waiver or alteration is requested:

- Provide the rationale for each.

The unit of evaluation for this study is the clinic room, not patients or subjects. However, we request waiver of informed consent and HIPAA authorization to access patient records during the study in order to record aggregate data. We will not collect or document individual patient information, as described in the next section. These data are necessary to determine if study rooms have been "exposed" to the same number and types of patients.

List the specific protected health information (PHI) to be collected and its source(s):

- (Note: PHI = health information + identifiers)

No PHI will be recorded or collected for this project. We will, however, we will briefly review the records of those patients to determine if they have an active infection or history of colonization or infection. These data will be aggregated (e.g., 1 patient out of 10 total for study room 1 on study day 1) and entered into our databases. No individual patient data will be recorded.

Criteria for Waiver: The DUHS IRB may waive the requirement for informed consent and authorization if all of the following criteria are met:

- Please respond to each item in the space below using protocol-specific language to provide justification:

a) The research or clinical investigation involves no more than minimal risk to subjects:

We believe the research involves no more than minimal risk, as the unit of evaluation is the clinic room. Patients who receive care in these rooms will have no increase in risk. The only potential risk would be loss of privacy. Howe ver, we will not record individual patient data or PHI. Thus, we believe the risk for loss of privacy is essentially zero.

b) The waiver or alteration will not adversely affect the rights and welfare of the subjects. Include a description of any measures to be taken to ensure that the rights and welfare of subjects will be protected:

A waiver will not adversely affect the rights and welfare of patients for the following reasons:

- The culture swabs will be obtained from the environment and not the patients.
- The non-PHI, aggregated data collected about the patients who receive care in study rooms will be kept on encrypted computers/databases. As such, this study poses minimal risk to patient privacy.

c) Whenever appropriate, the subjects will be provided with additional pertinent information after participation:

In the event that individual patients or staff members request information about the study, we will readily provide information and answer questions on the purpose of the trial, the procedures involved in the trial, and whom to contact for further information.

d) If this research activity relates to research involving deception, explain how subjects will be provided with additional pertinent information after study participation and what information will be provided. Otherwise indicate "not applicable":

Not applicable

e) The use or disclosure of protected health information involves no more than minimal risk to the privacy of individuals, based on, at least, the presence of the following elements (e1. and e2.)

Demonstrate that the use or disclosure of PHI involves no more than minimal risk to the privacy of subjects by describing the plans requested below:

e1) An adequate plan to protect the identifiers from improper use and disclosure.
Describe the plan (how protection will be accomplished) and indicate where the PHI will be stored and who will have access:

No PHI will be collected or recorded. PHI will only be accessed during evaluation for history of infection.

e2) An adequate plan to destroy the identifiers at the earliest opportunity consistent with conduct of the research, unless there is a health or research justification for retaining the identifiers or such retention is otherwise required by law.

Describe the plan (how and when identifiers will be destroyed and by whom). If there is a health or research justification for retaining the identifiers or such retention is otherwise required by law, provide the reason to retain identifiers:

No PHI will be recorded.

e3) Adequate written assurances that the protected health information will not be reused or disclosed to any other person or entity except (i) as required by law, (ii) for authorized oversight of the research study, or (iii) for other research for which the use or disclosure of PHI would be permitted by the HIPAA Privacy Rule. By electronically signing this submission, the PI provides this written assurance:

The PI has electronically signed this submission.

f) The research could not practicably be conducted or carried out without the waiver or alteration:

- Explain why informed consent/authorization can not be obtained from subjects.

The unit of evaluation and randomization is the patient rooms, not patients. Yet, it is necessary for us to gather minimal information about the number and types of patients who receive care in study rooms to determine if study rooms were “exposed” to the same type of patients. This study could not practicably be conducted without a waiver because all patients who receive care in study rooms need to be accounted for. Requirement of consent would lead to patient dropouts and limit our ability to compare across study arms.

g) The research could not practicably be conducted or carried out without access to and use of the protected health information:

Temporary access to PHI to obtain clinical information on the patients is critical to the study. No PHI or identifiers will be permanently recorded.

h) For research using biospecimens or identifiable information, the research could not practicably be carried out without access to and use of the protected health information:

No specimens will be obtained from patients. All specimens will be collected from the environment. Temporary access to PHI to obtain the clinical information on the patients of enrolled nurses is critical to the study. No PHI or identifiers will be permanently recorded.

Application Questions Complete

Please click Save & Continue to proceed to the Initial Submission Packet.

The Initial Submission Packet is a short form filled out after the protocol application has been completed. This is an area to attach protocol-related documents, consent forms, and review the application.

DukeHealth
Institutional Review Board

DUHS INSTITUTIONAL REVIEW BOARD NOTIFICATION OF EXEMPT AMENDMENT

Protocol ID: Pro00103576
Reference ID: Pro00103576-AMD-1.0
Principal Investigator: Daniel Sexton
Protocol Title: Measuring the Impact of an Enhanced Strategy for Daily Disinfection in Acute Care Hospital Rooms

The Duke University Health System Institutional Review Board for Clinical Investigations has conducted the following activity on the study cited above:
Activity: Amendment **Review Type:** Exempt
Review Date:
Issue Date: April 13, 2021
Expiration Date: 01/01/2100

Application: 1.1

This amendment represents a change that is in accordance with the previous IRB outcome determination.

This Declaration of Exemption from further IRB Review does not expire. However, changes to the proposed research will require an amendment requesting re-review for exemption. Reportable serious adverse events and unanticipated problems related to the research that place subjects or others at risk of physical, psychological, economic, or social harm must be promptly reported to the IRB and will result in reconsideration of the activity's exempt status.

The Duke University Health System Institutional Review Board for Clinical Investigations (DUHS IRB), is duly constituted, fulfilling all requirements for diversity, and has written procedures for initial and continuing review of human research protocols. The DUHS IRB complies with all U.S. regulatory requirements related to the protection of human research participants. Specifically, the DUHS IRB complies with 45CFR46, 21CFR50, 21CFR56, 21CFR312, 21CFR812, and 45CFR164.508-514. In addition, the DUHS IRB complies with the Guidelines of the International Conference on Harmonization to the extent required by the U. S. Food and Drug Administration.

DUHS Institutional Review Board
2424 Erwin Rd | Suite 405 | Durham, NC | 919.668.5111
Federalwide Assurance No: FWA 00009025

DUHS INSTITUTIONAL REVIEW BOARD DECLARATION OF ACTIVITY NOT MEETING THE DEFINITION OF RESEARCH

The DUHS IRB has determined that the following activity does not meet the definition of research as described in 45 CFR 46.102(d), 21 CFR 50.3(c) and 21 CFR 56.10(c) and satisfies the Privacy Rule as described in 45 CFR 164.514.

Protocol ID: Pro00103576
Reference ID: 332754
Protocol Title: Measuring the Impact of an Enhanced Strategy for Daily Disinfection in Acute Care Hospital Rooms
Principal Investigator: Daniel Sexton

This IRB declaration is in effect from August 29, 2019 and does not expire. However, please be advised that any change to the proposed research will require re-review by the IRB.

DUHS Institutional Review Board
2424 Erwin Rd | Suite 405 | Durham, NC | 919.668.5111
Federalwide Assurance No: FWA 00009025

Amended Protocol

Amended on 12/2/2021, Prior to any enrollment of presented study data – This was done after and in response to a pilot test of the original protocol.

Measuring the Impact of Enhanced Strategies for Daily Disinfection in Acute Care Hospital Rooms: Sani24 vs. Routine Daily Disinfection

Principal Investigator: Nicholas Turner, MD
Sub-Investigators: Bobby Warren, MPS

Research Plan

Objective: To determine the effectiveness of Sani24 in the "real world" hospital environment compared to routine disinfection.
Hypothesis: Daily application of Sani24 will lead to decreased environmental bioburden compared to routine daily disinfection.
Methods:
Design: randomized controlled trial
Outcomes:

- Primary outcome – Total Colony Forming Units (CFUs) on high touch surfaces in acute care hospital rooms over a 24-hour period
- Secondary outcomes
    - Change in CFU from baseline to 24 hours later
    - Total CFUs of epidemiologically important vegetative bacteria (MRSA, MSSA, VRE, MDR GN)
        - Proportion of samples with epidemiologically important vegetative bacteria
    - Change in CFU of epidemiologically important vegetative bacteria from baseline to 24 hours later
        - Change in proportion of samples with epidemiologically important vegetative bacteria
- Descriptive outcome – Patient perception of routine daily disinfection and intervention-disinfectant usage.

Approach:

- Inclusion criteria: Rooms with occupants on contact precautions
- Randomization: High touch surfaces will be randomized by right and left sides, 1:1 to intervention or control.
    - Following collection of baseline samples:
        - Intervention – team will apply Sani24 to high touch surfaces throughout room on day 0
        - Control – no change in routine daily disinfection
- Routine disinfection tracking
    - UV-C fluorescing gel dots will be applied to all randomized surfaces to ensure study disinfectant has not been removed from the surface
- Microbiological sampling:
    - Location:
        - 3 samples will be obtained for each study arm, one at each point of measurement: bedrails, overbed table, and sink

- Timing:
  - Day 0 - immediately before application of disinfectant (intervention) or similar time point (control)
  - Day 1 – 24 hours following the application of disinfectant (intervention) or similar time point (control)
- Measurement:
  - CFUs on high touch objects obtained via sponge and stomacher technique

- Patients will be asked a short series of questions on day 1 about routine daily disinfection and the disinfectant if in the intervention arm
  - Question examples:
    - "How well do you think your room is cleaned?"
    - "How well do you think your room is disinfected?"
    - "Do you have any concerns about the disinfectant being used?"

Study size: 50 patient rooms will be enrolled

- Each measured twice in three locations = 600 sponge samples

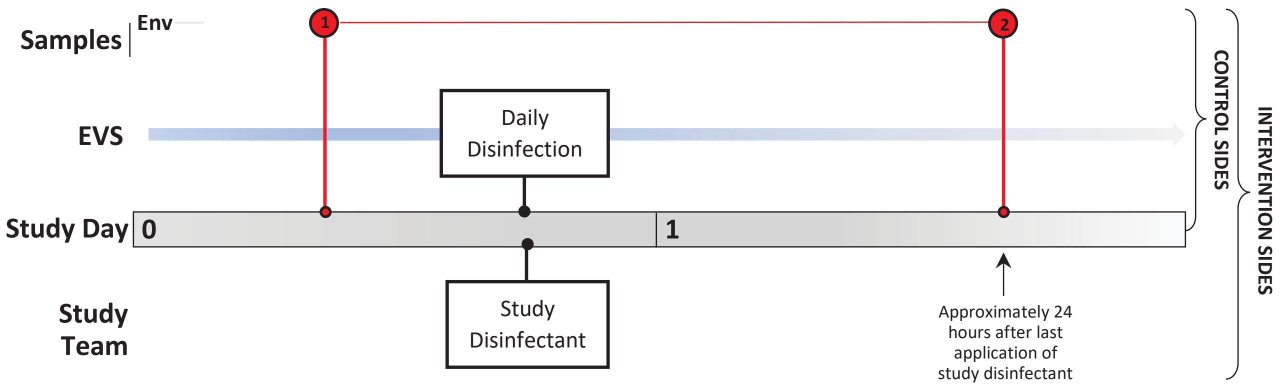

Figure 1. Study design.

Analysis:
Primary and secondary outcomes will be analyzed using a top-down approach.
Primary outcome – Total CFUs on high touch surfaces 24 hours after enrollment

- Total room CFU on day 1 for the entire study
- Total room CFU on day 1 compared between study arms
- Total room CFU on day 1compared within each couplet

- Sample area CFU on day 1 for the entire study
- Sample area CFU on day 1 compared between study arms
- Sample area CFU on day 1 compared within each couplet

CFU comparisons will be analyzed using the Wilcoxon rank sum test, proportions with the proportion Z score test and categorical variables with the Chi-squared test. A p-value of 0.05 will be considered significant and all statistical tests will be 2-tailed. All testing will be completed using R software (R Foundation for Statistical Computing, Vienna, Austria).

References

1. Magill SS, Edwards JR, Bamberg W et al. Multistate point-prevalence survey of health care-associated infections. 2015;370(13):1198-1208. doi:10.1056/NEJMoa1306801.Multistate

2. PW S. Economic burden of healthcare-associated infections: An American perspective. Expert Review of Pharmacoeconomics & Outcomes Research. 2009;9:1-10. doi:10.1586/erp.09.53.Economic

3. Anderson DJ, Moehring RW, Weber DJ, et al. Effectiveness of targeted enhanced terminal room disinfection on hospital-wide acquisition and infection with multidrug-resistant organisms and Clostridium difficile: a secondary analysis of a multicentre cluster randomised controlled trial with crossover design (BETR Disinfection). *Lancet Infect Dis*. 2018;18(8):845-853. doi:10.1016/S1473-3099(18)30278-0
