# Supplementary material for: An Enhanced Strategy for Daily Disinfection in Acute Care Hospital Rooms: A Randomized Clinical Trial
Source: JAMA Netw Open. 2022 Nov 15;5(11):e2242131. doi: 10.1001/jamanetworkopen.2022.42131 (PMC9667331; doi:10.1001/jamanetworkopen.2022.42131)
Supplement: Supplement 2. — Data Sharing Statement [file jamanetwopen-e2242131-s002.pdf]

Data Sharing Statement

Warren. An Enhanced Strategy for Daily Disinfection in Acute Care Hospital Rooms. *JAMA Netw Open*. Published November 15, 2022. doi:10.1001/jamanetworkopen.2022.42131

Data
Data available: No

Additional Information
Explanation for why data not available: The data of this article and study will not be shared.
